# Role for Heat Shock Protein 90α in the Proliferation and Migration of HaCaT Cells and in the Deep Second-Degree Burn Wound Healing in Mice

**DOI:** 10.1371/journal.pone.0103723

**Published:** 2014-08-11

**Authors:** Yue Zhang, Xiaozhi Bai, Yunchuan Wang, Na Li, Xiaoqiang Li, Fei Han, Linlin Su, Dahai Hu

**Affiliations:** Department of Burns and Cutaneous Surgery, Xijing Hospital, Fourth Military Medical University, Xi'an, Shannxi, China; University of Tennessee, United States of America

## Abstract

Inflammation, proliferation, and tissue remodeling are essential steps for wound healing. The hypoxic wound microenvironment promotes cell migration through a hypoxia—heat shock protein 90 alpha (Hsp90α)—low density lipoprotein receptor-related protein-1 (LRP-1) autocrine loop. To elucidate the role of this autocrine loop on burn wound healing, we investigated the expression profile of Hsp90α at the edge of burn wounds and found a transient increase in both mRNA and protein levels. Experiments performed with a human keratinocyte cell line—HaCaT also confirmed above results. 17-dimethylaminoethylamino-17demethoxygeldanamycin hydrochloride (17-DMAG), an Hsp90α inhibitor, was used to further evaluate the function of Hsp90α in wound healing. Consistently, topical application of Hsp90α in the early stage of deep second-degree burn wounds led to reduced inflammation and increased tissue granulation, with a concomitant reduction in the size of the wound at each time point tested (*p*<0.05). Consequently, epidermal cells at the wound margin progressed more rapidly causing an expedited healing process. In conclusion, these results provided a rationale for the therapeutic effect of Hsp90α on the burn wound management.

## Introduction

There are three key phases in wound healing: inflammation, proliferation, and tissue remodeling [1]. The migration of epidermal and dermal cells to the wound bed is regulated by extracellular matrix and soluble growth factors, which is critical to would healing [2], [3]. Clinicians have made great progress in the management of wound healing by using genes, cytokines, chemokines, and surgery as therapeutic strategies [1], [4], [5], [6]. However, additional investigation is largely needed to understand the complex pathogenic mechanism involved in burn wound healing and thus reduce expenditure and shorten time cost related to this course.

In the burn area, residual skin cells secret growth factors such as vascular endothelial growth factor (VEGF) [7] and platelet-derived growth factor (PDGF) [8]. These cytokines are widely accepted as the driving force behind wound healing [9]. Research has also revealed that among these cytokines only recombinant human platelet-derived growth factor-BB (rhPDGF-BB) is effective in promoting wound healing, however, the high cost of treatment as well as the high risk of cancer induction have greatly limited its clinical use [9], [10]. Additionally, transforming growth factor-beta 3 (TGF-β3) is widely expressed in various tissues and can inhibit the wound-healing effects of PDGF-BB and VEGF, thus these cytokines alone are not sufficient to promote the migration of dermal fibroblasts (DFs) or human dermal microvascular endothelial cells (HDMECs) [11], [12], [13]. Therefore, the mechanism driving the skin cell migration to the wound bed when TGF-β3 is abundantly expressed in the same tissue needs to be elucidated.

Li and colleagues have taken initial steps toward addressing this issue [14]. They have found that Hsp90α was responsible for the migration of human epidermal and dermal fibroblasts [14]. Hsp90α is a chaperone protein induced by environmental stresses and widely present in various mammalian cells, where it regulates cell homeostasis. Once injury, the integrity of vessel would be disrupted, and cells began to consume oxygen rapidly thus creating a hypoxic microenvironment [15], [16], [17], which then induced Hsp90α secretion mediated by hypoxia-inducible factor-1 alpha (HIF-1α) and in turn stimulated skin cell migration via LRP-1 to promote wound healing [18], [19], [20].

Despite the proposed role of Hsp90α in wound healing, details on its mechanism of action are still poorly understood. Moreover, there is no systematic exploration to the relationship between burn injury and Hsp90α expression. In the present study, we investigated the role of Hsp90α on would healing in burn injuried mice. We hypothesize that the increased production of Hsp90α at the burn site might improve wound healing. To illustrate the role of Hsp90α in burn injury, we determined the change of Hsp90α expression over time in mice with deep second-degree burns. We then evaluated the effect of Hsp90α on wound healing elicited by creating a scratch in heat-shocked immortalized human keratinocytes—HaCaT cells, an *in vitro* human skin equivalent model. The use of HaCaT cells avoids inter-individual differences of primary cells and limites the expansion capacity of keratinocytes, in the meantime maintains the epidermal differentiation and strong proliferation capacity [21], [22], [23], [24]. Next, we performed recovery assay in the presence of Hsp90α and/or 17-DMAG, a putative Hsp90α inhibitor. We also assessed the difference in wound size as well as the regeneration of epidermal areas among groups of mice exposed to each substance.

## Materials and Methods

### Ethics Statement

All experimental procedures were conducted under the protocol reviewed and approved by Institutional Ethical Committee of the Fourth Military Medical University.

### Deep Second-Degree Burns in Mice

**Figure 1:**
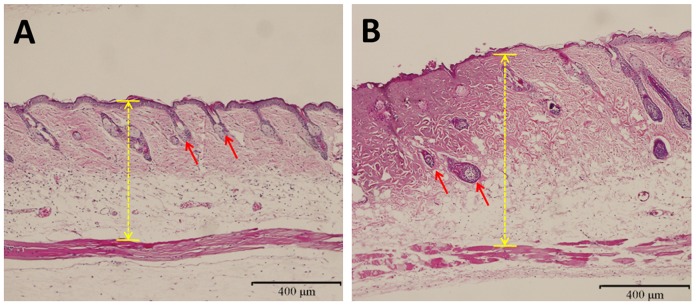
HE staining showing the histological changes of mouse skin after deep second-degree burn injury. (**A**) Normal skin. (**B**) Burned skin from mice suffering from hot steam for 4 s. Deep partial-thickness burn injury was observed and hair follicles were not damaged. Yellow arrowed lines indicated the thickness of skin in unburned and burned mice. Red arrows indicated hair follicles.

Six-week-old male Balb/c mice (n = 30, weighing 20 g each) were purchased from Animal Research Center of Fourth Military Medical University. Mice were housed under standard laboratory conditions at 12 h-light/12 h-dark at 25°C with food and water supplied daily. Mice were first anesthetized by intraperitoneal (i.p.) injection of pentobarbital sodium (60 mg/kg b.w.), the hair on the back and flank was clipped and depilated with 10% Na_2_S (dissolved in alcohol). The whole area was then thoroughly rinsed with distilled water. 24 h later, dorsal skins of mice were exposed to hot steam at 98°C for 4 s to create deep second-degree burns. The burn diameter was 20 mm, and the depth was further confirmed by observing the pathological change on burned tissue section (Fig. 1).

To optimize the hot steam exposure time for deep second-degree burn injury, 40 mice were randomly divided into four groups and exposed to hot steam for 2, 4, 6 or 8 s, respectively. 5 mice randomly selected from each group were sacrificed 12 h later, the remaining 5 mice in each group were sacrificed 24 h later. Samples were cut and fixed in formalin overnight, then subjected to hematoxylin and eosin (HE) staining to determine the optimal steam exposure time as well as to confirm the deep second-degree burn.

### Real-Time PCR Analysis

Mice used for real-time PCR experiment were sacrificed at 0, 0.5, 1, 3, 6, 12 and 24 h post-burns. Full-thickness burns were excised from the wound edge. Total RNA was extracted with Trizol reagent (Invitrogen, CA, USA), and 2 µg RNA was reverse transcribed by using SuperScript and PrimeScript RT Reagent Kit (TaKaRa, Dalian, China). Subsequently, real-time PCR was conducted by Bio-Rad iQ5 real-time PCR detection system (Bio-Rad Laboratories Inc, CA, USA). The cDNAs of mouse Hsp90α and glyceraldehyde-3-phosphate dehydrogenase (GAPDH) were detected using the following primer pairs: 5′-CCATGCTAACAGGATCTACAGGA-3′, 5′-TCTTCAGTTACAGCAGCACTGG-3′ and 5′-TGTGTCCGTCGTGGATCTGA-3′, 5′-TTGCTGTTGAAGTCGCAGGAG-3′, respectively. The mRNA level of Hsp90α was normalized against that of GAPDH.

### Western Blot Analysis

Mice used for western blot analysis were sacrificed at 0, 6, 12, 24, 48 and 72 h post burns. Total protein from burn wounds were obtained using lysis buffer supplemented with complete protease inhibitor cocktail (Beyotime, China). A micro-BCA protein assay kit (Beyotime, China) was used to measure protein concentration in each sample. Total protein was then separated by sodium dodecyl sulfate-polyacrylamide gel electrophoresis (SDS-PAGE) and transferred to PVDF membrane (Millipore, Bedford, MA, USA), which was further incubated with rabbit anti-Hsp90α monoclonal antibody (1∶1000 dilution, Epitomic, USA) or rabbit anti-β-actin monoclonal antibody (1∶1000 dilution, Cell Signaling, USA) at 4°C overnight, followed by incubation with goat anti-rabbit IgG secondary antibody (1∶3000, ZSGB-BIO, Beijing, China). Protein bands were visualized by FluorChem FC digital imaging system (Alpha Innotech).

### Immunohistochemistry

Mouse skin tissue containing both wound and unwound areas was stained with Hsp90α mAb (1∶100 dilution, Epitomics, California, USA). After paraffin section rehydration, blocking, and antibody incubation, diaminobenzidine (DAB) was used as the chromogen to visualize Hsp90α positive staining, while hematoxylin was used for counterstaining of nuclei. Images were captured by an FSX100 microscope (Olympus, Japan).

### *In vitro* HaCaT Cell Culture

HaCaT, a human immortalized keratinocyte cell line, was purchased from China Center for Type Culture Collection (Wuhan, China). Cells were grown in Dulbecco's modified Eagle's medium (DMEM) supplemented with 10% fetal bovine serum (Gibco, Grand Island, NY, USA), 100 U/ml penicillin and 100 U/ml streptomycin. Cells were cultivated in an incubator at 37°C.

### Heat Shock Treatment on HaCaT Cells

*In vitro* heat shock assay was conducted according to the procedure described by Ting-Ting Wang [25]. Briefly, HaCaT cells were seeded on six-well tissue culture plates and then placed in a water bath at 45°C for exact 15 minutes.

### Scratch Assay

A straight scratch line was made on HaCaT cells with a p200 pipet tip after heat-shock treatment, and cells were washed with PBS and further cultured in DMEM containing 10% FBS. Then cells were treated with saline, 8 µg/ml recombinant Hsp90α protein (Cayman Chemical, USA) or 0.5 µmol/L 17-DMAG (InvivoGen, Santiago, USA). Images were taken at 0, 6, 12 and 24 h post-treatment. 17-DMAG, a water-soluble derivative of geldanamycin analogue, can inhibit Hsp90α function [26] and its cellular toxicity was tested first by MTT assay (shown in Fig. S1), and we used a low dose of 0.5 µg/ml in our experiments. Gap width of the scratch was measured and recorded, and then compared to the initial gap size at 0 h.

### Flow Cytometry Analysis

Cell cycle distribution was analyzed by flow cytometry (Facsaria, BD, USA). HaCaT cells were evenly divided into three groups receiving differential treatments of saline, 8 µg/ml Hsp90α or 0.5 µmol/L 17-DMAG for 24 h, and then placed in a water bath at 45°C for 15 min, followed by an incubation at 37°C for 6 h. Cells were then harvested and rinsed with PBS, fixed with 95% ([v]/[v]) ice-cold ethanol and resuspended in staining buffer. DNA contents of stained nuclei were analyzed, and cell numbers in different cell cycle phases were calculated.

Cell apoptosis was determined at the same time as analysis of cell cycle distribution. After cells were harvested, fixed and washed, the binding buffer containing FITC-Annexin V and propidium iodide (PI) was added to cells. The mixture was then incubated in dark at room temperature for 15 min. Apoptotic cells were detected by flow cytometry (BD, Facsaria, USA).

### *In vivo* Wound-Healing Assay

Six-week-old male Balb/c mice (n = 30, weighing 20 g each) were suffered from hot steam for 4 s and then randomly divided into three groups receiving differential local applications of saline, Hsp90α or i.p. injection of 17-DMAG before burn injury. Pre-treatment of mice with 17-DMAG was performed as described by Hackl C and the colleagues [27]. In short, mice received i.p. injection of 17-DMAG at 25 mg/kg b.w., three times per week, and was subject to deep second-degree burns two weeks after the initial dose. The inhibitory effect of 17-DMAG on Hsp90α was dertermined by the induction of Hsp70 expression [28]. Images were taken at 0, 5, 9, 13 and 21 days after burns by using Canon EOS 50D digital camera, and wound surface area was calculated with Image-Pro Plus software (Media Cybernetics). [(wound area on day x – wound area on day 0)/wound area on day 0×100] is defined here as the relative amount of wound closure [29].

### Tissue Biopsy Harvesting

Mice were sacrificed 7 days post burn injury. Full-thickness skin and underlying adherent tissues were excised and fixed in formalin overnight, and then subject to HE staining. Newly healed wounds showed a flat layer of re-epithelialized hair follicle-free epidermis, while unwound skins were full of hair follicles at the dermal-epidermal junction. The average re-epithelialized gap was defined as the distance between the advancing edges of epidermal keratinocyte migration. The assessment of wound contraction was based on the average distance in reference to the initial size of the wound [18]. All images were captured by a FSX100 microscope (Olympus, Japan), and multiple overlapping pictures were used to reconstitute the entire wound. Alpha Ease FC version 4.1.0 (Alpha Innotech Corporation, IL), an image analysis software, was used for open wound healing analysis, and Student's *t*-test was used for statistical analysis [18].

### Data Analysis

All data, with the exception of wound measurements, are presented as means±SD. Wound measurements are presented as means±SEM. Differences between experimental and control groups were determined by one way ANOVA testing. *p*<0.05 was considered statistically significant. All statistical analyses were performed with SPSS 13.0 software.

## Results

### Evaluation of Deep Second-Degree Burns in Mice

The authenticity of deep second-degree burn was confirmed by histological evaluation on HE-stained burn wound sections. A deep partial-thickness burn injury was observed in mice treated with hot steam for 4 s. As shown in Fig. 1, structures of the epidermis and a deeper part of the dermis were partially damaged, additionally, damaged cells could not be identified (as indicated by arrows in Fig. 1B). However, hair follicles in hot stream treated skin still looked normal.

### Enhanced Expression of Hsp90α in Burned Skin

**Figure 2:**
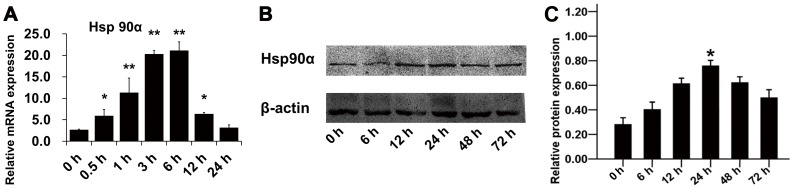
RT-PCR and IB assays showing the changes on Hsp90α expression after burn injury at the wound edge in mice. (**A**) mRNA level of Hsp90α was measured at each time point after burn injury by real-time PCR and normalized against GAPDH. Results showed that the mRNA level in burned skin peaked at 3∼6 h and was 20 times higher than that in control mice (*p*<0.05). (**B**) The change of Hsp90α protein level after burn injury was analyzed by western blotting (*p*<0.05). (**C**) Histogram quantified the relative Hsp90α level in (**B**).

**Figure 3:**
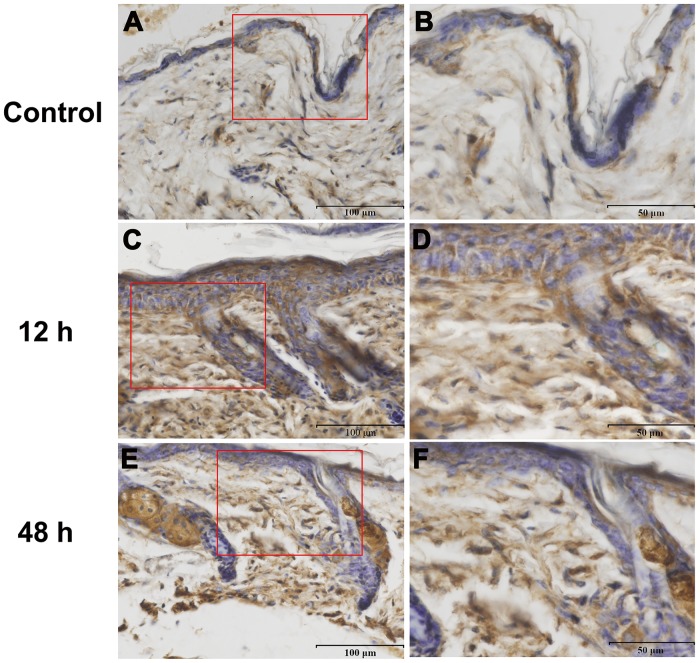
IHC assay showing the changes of Hsp90α immunostaining in burned mouse skin. (**A**) Unwound normal skin showed a few positive Hsp90α stainings. (**C**, **E**) Epidermal and dermal tissues after burn injury appeared more positive brownish stainings, indicating that Hsp90α was induced after the burn stimulation. In addition, Hsp90α level was the highest at 12 h post-treatment (**C**) and somewhat decreased at 48 h (**E**). Magnification of red boxed areas in (**A**), (**C**) and (**E**) was shown as (**B**), (**D**)and (**F**), respectively.

To determine whether a correlation existed between burn injury and Hsp90α expression, we took three approaches to assessing the change on Hsp90α expression after burn injury. Firstly, real-time PCR analysis was used to assess the transcriptional level of Hsp90α during early stages of wound healing. Results indicated that Hsp90α mRNA level in burned skin peaked at 3∼6 h and were 20 times higher than that in unburned mice (Fig. 2A, n = 3 for each time point, *p*<0.05). Secondly, Hsp90α protein level was increased significantly from 12 h after burn injury and maintained high till 72 h as shown in Fig. 2B. These results provided direct evidences that burn injury could stimulate wound edge area to produce more Hsp90α at both mRNA and protein levels. Previous studies on the function of Hsp90α in wound healing suggested that Hsp90α was more likely to be secreted out to the extracellular space, where it exerted important biological functions [30]. Thirdly, immunohistochemistry was performed to visualize the change on Hsp90α expression after burn treatment (Fig. 3). The burned area showed a high density of Hsp90α immunoreactive staining while the control had less Hsp90α expression. The contrast was most notable at 12 h after burns, with a slight reduction of Hsp90α level at 48 h.

### Hsp90α Accelerates the Migration of HaCaT Cells in an *in vitro* Heat Shock Model

**Figure 4:**
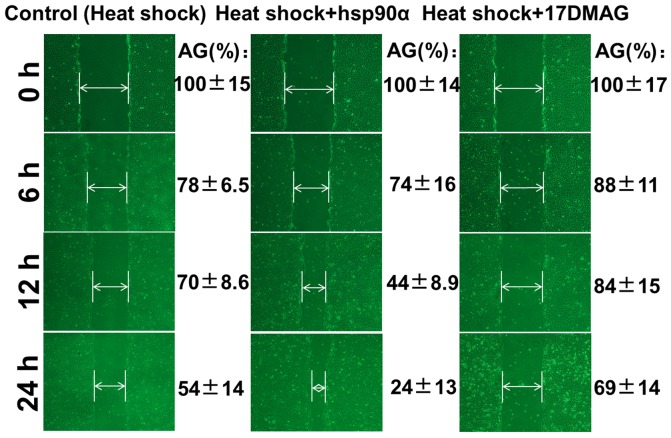
An *in vitro* scratch assay showing the effects of Hsp90α on the migration of heat-shocked cells. Images were taken at the indicated time of incubation. Hsp90α-treated group showed more rapid reduction in the gap size at each time point tested than that in the control group, while 17-DMAG group showed slower gap closure even than the control (*p*<0.05). AG, average gap, normalized to the gap size at 0 h.

We used an *in vitro* assay to determine whether Hsp90α could enhance the skin cell migration in a cell-based heat shock model. A slow reduction in the gap of the scratch was observed in control group, and the gap shrinked to approximately 50% of the initial size 24 h later. Hsp90α-added group showed a more rapid reduction in gap size, and the gap was almost closed 24 h later. While 17-DMAG-treated group showed a slower gap closure even than the control (Fig. 4). These findings suggested that Hsp90α could stimulate skin cell migration in the *in vitro* heat shock model.

### Hsp90α Promotes HaCaT Cell Viability and Inhibites Its Apoptosis

**Figure 5:**
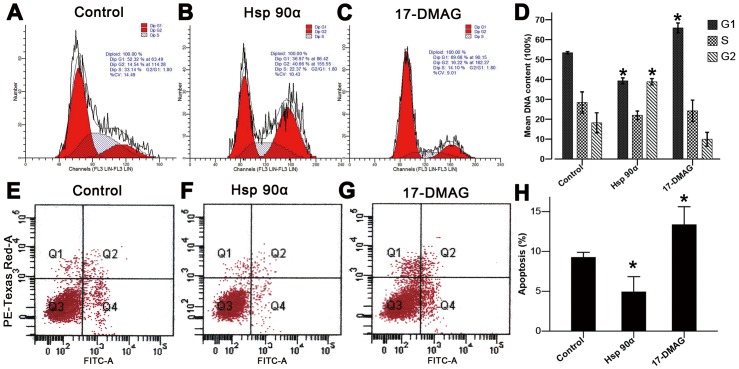
Flow cytometry assay showing the effects of Hsp90α on cell cycle progression and apoptosis following heat shock. Heat-shocked cells treated with (**A**) saline, (**B**) Hsp90α, and (**C**) 17-DMAG for 24 h were assessed by flow cytometry. Hsp90α increased the number of cells in G1 phase, while 17-DMAG induced cell cycle arrest at G0–G1 phases. These results were represented histogramatically in (**D**). FITC- Annexin V/propidium iodide (PI) was used to measure the apoptosis rate of HaCaT cells following three treatments: (**E**) saline, (**F**) Hsp90α, and (**G**)17-DMAG. The percentage of apoptotic cell population was decreased in Hsp90α group and increased in 17-DMAG group (**H**) (*p*<0.05).

Hsp90α is known to function as a chaperone for a variety of proteins that regulate cell cycle progression and apoptosis [27], [28]. To determine the effect of Hsp90α on the viability of heat-shocked cells, we evaluated the cell cycle distribution and the level of HaCaT cell apoptosis by flow cytometry. As shown in Fig. 5A–D, heat-shocked cells treated with Hsp90α exhibited an obvious decrease in the cell number of G1 phrase, while 17-DMAG showed an inductive effect on G1 cell cycle arrest. Furthermore, as shown in Fig. 5E–H, the apoptosis rate of HaCaT cells was significantly decreased after Hsp90α treatment, while 17-DMAG treatment showed the opposite effect. These results further illustrated the role of Hsp90α on the viability and apoptosis of heat-shocked cells.

### Local Application of Hsp90α Improves Burn Wound Healing *in vivo*

**Figure 6:**
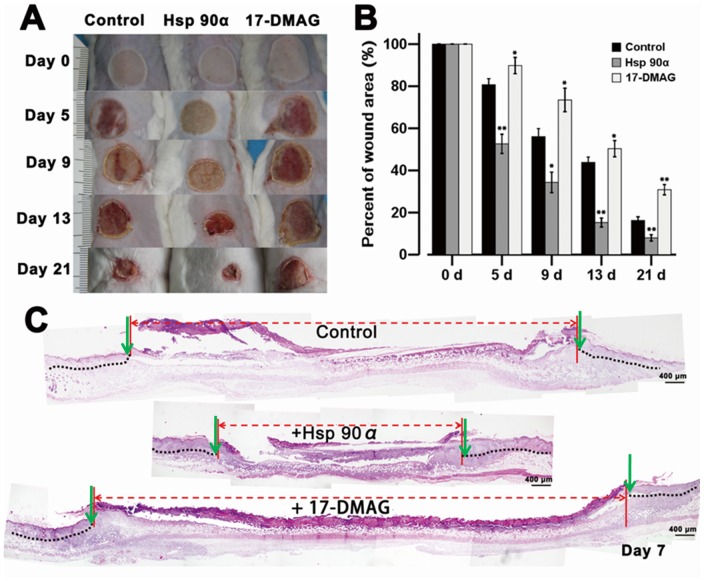
An *in vivo* study showing the effects of Hsp90α on burn wound healing. (**A**) Deep second-degree burn wounds (20 mm in diameter) on Balb/c mice were treated topically with saline or Hsp90α (1 µg/ml) daily for 5 days following burn injury. In 17-DMAG group, 17-DMAG at 25 mg/kg b.w. was injected intraperitoneally three times per week for two weeks prior to burn treatment, and then saline was applied topically. (**B**) Histogram quantified the wound size on day 0, 5, 9, 13 and 21 (n = 5 in each group). (**C**) Hsp90α promoted the re-epithelialization of deep second-degree burn wound. On day 7 after burn injury, biopsies of burned and unburned skins were excised from control, Hsp90α, and 17-DMAG-treated groups. Samples were then HE stained and photographed with an FSX100 microscope. Images were reconstituted to show the whole healed and unhealed areas. Area between two vertical red lines indicated unhealed skin, while area with black dotted lines indicated newly formed epidermis. Green arrows marked the advancing migrating epithelial tongues on each side of the wounds.

To examine the therapeutic potential of Hsp90α on burn wound healing, microscopic and histological experiments were performed. Highly purified human recombinant Hsp90α protein was dissolved at 1 µg/ml in saline, and locally applied to burn wounds daily for the first 5 days following burn injury. As shown in Fig. 6A, the wound size in control group was slowly reduced each day. In Hsp90α-treated group, the process of wound healing was more rapid, with gradually smaller wound each day (*p*<0.05). While mice pre-treated with 17-DMAG showed an slower response on wound healing even than the control. The degree of edema and hyperemia in Hsp90α group was also less pronounced than that in other two groups. Wound size was measured on day 0, 5, 9, 13 and 21 (Fig. 6B, *p*<0.05). Results suggested that Hsp90α could significantly accelerate wound closure and shorten the time course of healing.

In addition, on day 7 post-burn injury, biopsy specimens were excised from burn wounds and subjected to HE staining to determine the re-epithelialization and wound contraction under each treatment. As shown in Fig. 6C, Hsp90α group showed an enhanced reduction in the average re-epithelialized gap; moreover, inflammation was reduced, granulation tissue showed significant growth, and epidermal cells at wound margins progressed more rapidly than in other two groups. While the 17-DMAG group displayed the opposite effect. Taken together, results above indicated that Hsp90α could promote burn wound healing through accelerated re-epithelialization and granule tissue formation.

## Discussion

The mechanism under burn wound healing is a complicated network quite different from that in other types of wound such as incision, laceration or chronic ulcer with little tissue loss or interference with blood supply. The body could be compromised when skin was gradually lost and blood supply was not preserved after burn injury [31]. During the past decade, studies have investigated the effect of Hsp90α on wound healing [30], however, the precise role of Hsp90α in burn wound healing remains ambiguous.

Hsp90α, induced by heat, alcohol, UV radiation, oxidant and other stresses, plays an important role in tissue repair. It is one of the most abundant molecular chaperones and can maintain cell stability, assist the correct folding of nascent proteins, redirect misfolded proteins, and cause the degradation of a diverse set of client proteins [28]. Interestingly, Li and his colleagues found that Hsp90α, secreted by human skin cells at the wound edge, had a positive effect on driving the wound closure when purified Hsp90α was applied to the wound [32]. In addition, Hsp90α was found to be secreted in exosomes rather than by conventional transportation through the endoplasmic reticulum and Golgi apparatus [33].

TGF-β was widely expressed in burn wounds and could suppress dermal cell migration in skin wounds, with its level fluctuating during the first 15 days after deep second-degree burn injury [34]. Therefore, the migration of human epidermal cells is prior to DFs and HDMECs during wound healing. However, unlike many other growth factors, Hsp90α could override TGF-β-induced inhibitory effects and stimulate the migration of both DFs and HDMECs [35]. Thus, Hsp90α is considered as a suitable candidate for therapeutic application to overcome TGF-β-mediated inhibition of burn wound healing.

Many factors can influence the complex process of burn wound healing. After burn infliction, cells at the wound margin were more likely to undergo hypoxia [36], which was observed at the healing margin of the burn wound beginning at 48 h and peaking on day 3 [37] and played an important role on the activation of HIF-1 and the promotion of angiogenesis and vasculogenesis during wound healing [26], [38]. In response to hypoxia, human keratinocytes secreted Hsp90α via an HIF-1-dependent pathway, which in turn stimulated skin cell migration via LRP-1 [19], [32].

HIF-1, a physiological regulator of oxygen, played an essential role in skin cell migration in response to hypoxia by inducing Hsp90α secretion [18]. In our studies, Hsp90α mRNA level in burned mice was found to be 20 times higher than that in normal mice at 12 h post-burn injury, and its protein level was also modulated, however, the time course of its protein induction was delayed when compared to its mRNA induction. This was consistent with the results of immunohistochemistry. Thus, the dynamic regulation of Hsp90α level throughout the course of burn injury was consistent with its role in HIF-1-associated would healing process.

Hsp90α was known to promote skin cell migration into the wound region, where cells formed new connective tissues and blood vessels in remodeled skin [14], [39]. Here we used a heat-shocked *in vitro* scratch assay simulating *in vivo* burn injury to assess the function of Hsp90α in skin cell migration to the burn wound. In Hsp90α-treated group, the scratch gap almost closed after incubation for 24 h, whereas 17-DMAG impeded the closure process of the scratch. Interestingly, when we made a scratch first and then heat shocked HaCaT cells, we found that there was no obvious difference in the rate of gap closure between scratching first or heat shocking first as shown in Fig. S2. Considering that mice were burned first in *in vivo* experiment, we chose to heat shock cells first. Thus our study indicated that Hsp90α could accelerate the migration of heat-shocked skin cells, while blocking its action by 17-DMAG could diminish this facilitative effect. 17-DMAG theoretically retains the capacity to bind Hsp90 and inhibits the ATPase activity of Hsp90 in an essentially identical fashion [26]. Besides, to analyze the effect of Hsp90α on the regulation of cell cycle and apoptosis, additional studies using flow cytometry were conducted. Results showed that Hsp90α had positive effects on the growth of heat-shock cells, inducing cell proliferation and reducing apoptosis. However, 17-DMAG exposure was found to suppress the effect of Hsp90α on cell proliferation while induce apoptosis [26], [40], [41]. These findings are in consistent with the role of Hsp90α in enhancing burn regeneration process. Since Hsp90α had both effects on promoting cell migration and proliferation, results from the present *in vitro* scratch assay showed comprehensive effects of Hsp90α on cell migration and proliferation. Indeed, when mitomycin C was first added to HaCaT cells to inhibit the cell proliferation, Hsp90α group still showed more rapid reduction in the gap size at each time point than control group as shown in Fig. S3. In addition, when HaCaT cells were seeded on fibronectin-coated plates, the scratch assay showed the exact same results (shown in Fig. S4) as when no matrix was added.

Our data indicated that Hsp90α level peaked 1∼2 days after burn injury and then dropped off gradually. The therapeutic regimen of Hsp90α for deep second-degree burn in this experiment started as early as the first day after burn infliction and lasted for 5 days. Results showed that the wound size was significantly smaller in mice treated with Hsp90α than that in control group on all days tested, and also Hsp90α treatment accelerated wound healing at each time point and no adverse effects were observed. While the reverse effects were observed in 17-DMAG-treated group. Moreover, the re-epithelialization and skin wound contraction observed on day 7 by HE staining further verified these findings. Taken together, this study demonstrated the important role of Hsp90α in accelerating cell migration, proliferation and wound healing after burns injury. It also suggested that targeting Hsp90α during wound healing may be of therapeutic value in the treatment of deep second-degree burn.

## Supporting Information

Figure S1 **MTT assay evaluating the cellular toxicity of 17-DMAG on HaCaT cells.** Results showed that 17-DMAG at 0.5 µg/ml was a safe dose that would not induce cell death.
(TIF)

Figure S2 **An** ***in vitro*** **scratch assay showing the effect of Hsp90α on cell migration when the scratch was made first.** Cells were first scratched, then subjected to heat shock, and then received saline, Hsp90α or 17-DMAG treatment. Images were taken at the indicated time of incubation. Hsp90α group showed more rapid reduction in gap size at each time point than control group, while 17-DMAG group showed even slower gap closure than the control (*p*<0.05).
(TIF)

Figure S3 **An** ***in vitro*** **scratch assay showing the effect of Hsp90α on cell migration when mitomycin C was first added to inhibit the cell proliferation.** Images were taken at the indicated time of incubation. Hsp90α group also showed more rapid reduction in the gap size than the control group, while 17-DMAG group also showed slower gap closure than the control (*p*<0.05).
(TIF)

Figure S4 **An** ***in vitro*** **scratch assay showing the effect of Hsp90α on cell migration when fibronection was coated on the plates as the cell matrix.** Images were taken at the indicated time of incubation. Hsp90α group showed more rapid reduction in the gap size than the control group, while 17-DMAG group showed slower gap closure than the control (*p*<0.05).
(TIF)
