# Supplementary figures and images for: Role for Heat Shock Protein 90α in the Proliferation and Migration of HaCaT Cells and in the Deep Second-Degree Burn Wound Healing in Mice
Source: PLoS One. 2014 Aug 11;9(8):e103723. doi: 10.1371/journal.pone.0103723 (PMC4128658; doi:10.1371/journal.pone.0103723)

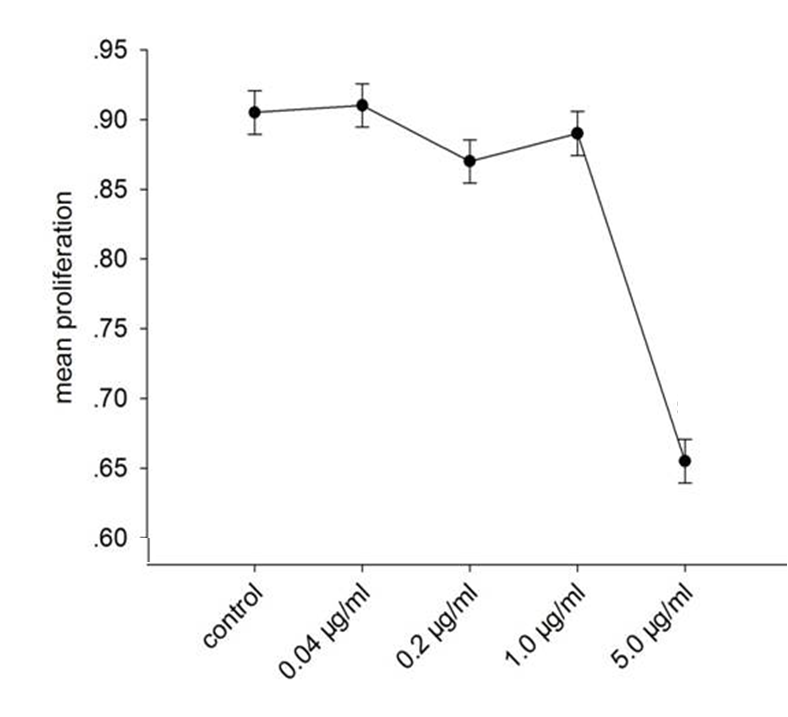

Supplement: Figure S1 — MTT assay evaluating the cellular toxicity of 17-DMAG on HaCaT cells. Results showed that 17-DMAG at 0.5 µg/ml was a safe dose that would not induce cell death. (TIF) [file pone.0103723.s001.tif]

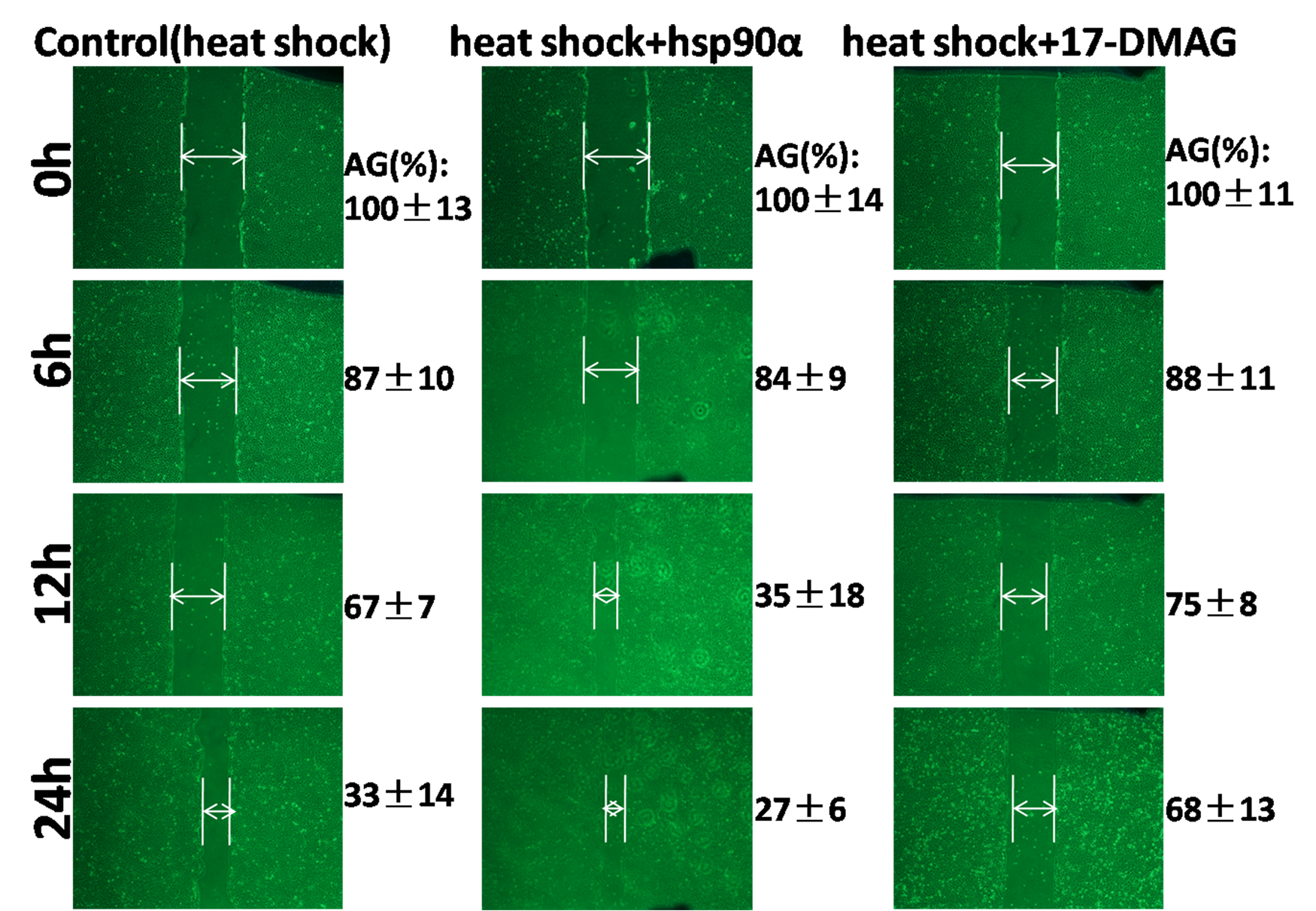

Supplement: Figure S2 — An in vitro scratch assay showing the effect of Hsp90α on cell migration when the scratch was made first. Cells were first scratched, then subjected to heat shock, and then received saline, Hsp90α or 17-DMAG treatment. Images were taken at the indicated time of incubation. Hsp90α group showed more rapid reduction in gap size at each time point than control group, while 17-DMAG group showed even slower gap closure than the control (p<0.05). (TIF) [file pone.0103723.s002.tif]

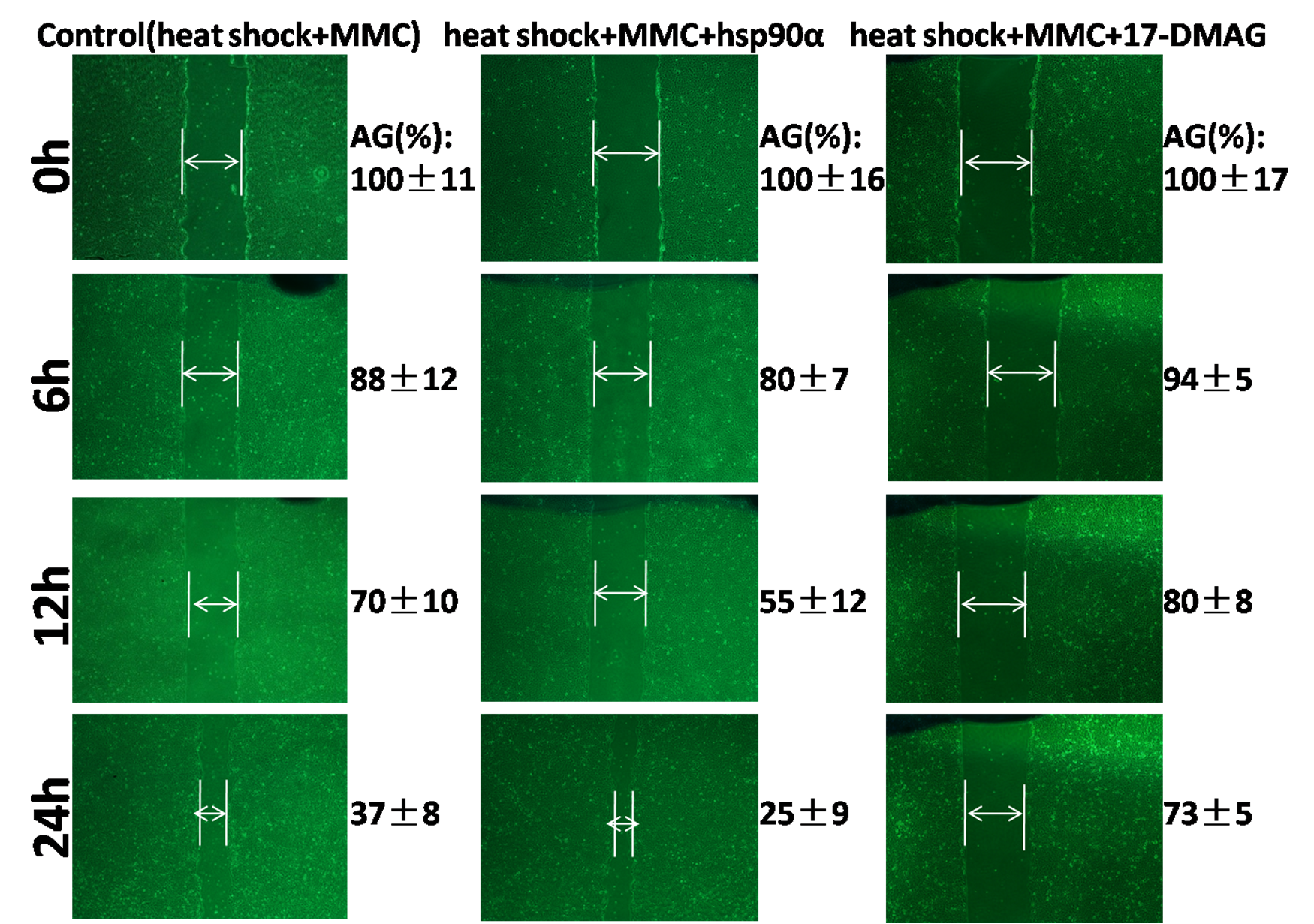

Supplement: Figure S3 — An in vitro scratch assay showing the effect of Hsp90α on cell migration when mitomycin C was first added to inhibit the cell proliferation. Images were taken at the indicated time of incubation. Hsp90α group also showed more rapid reduction in the gap size than the control group, while 17-DMAG group also showed slower gap closure than the control (p<0.05). (TIF) [file pone.0103723.s003.tif]

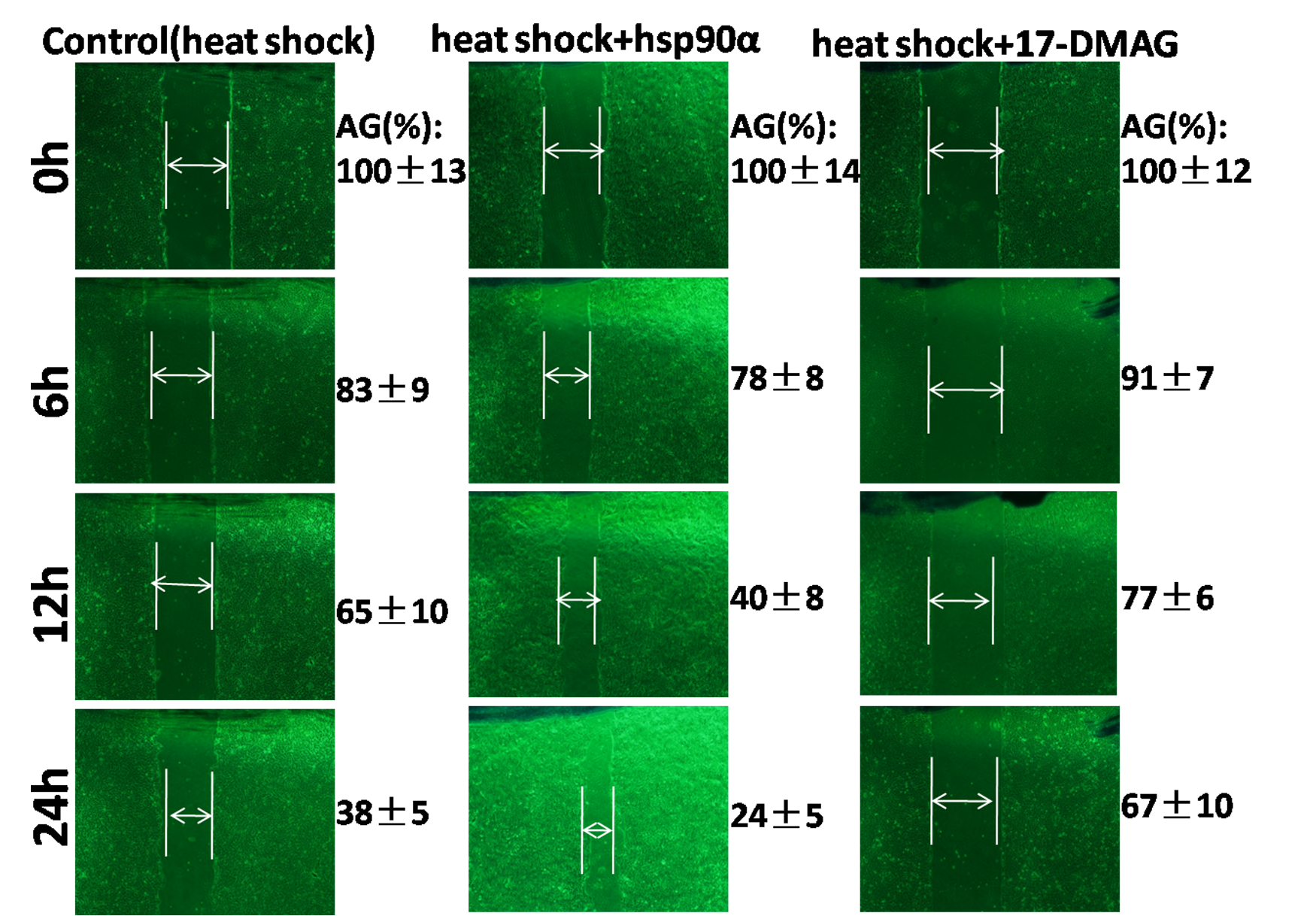

Supplement: Figure S4 — An in vitro scratch assay showing the effect of Hsp90α on cell migration when fibronection was coated on the plates as the cell matrix. Images were taken at the indicated time of incubation. Hsp90α group showed more rapid reduction in the gap size than the control group, while 17-DMAG group showed slower gap closure than the control (p<0.05). (TIF) [file pone.0103723.s004.tif]
